# Mapping of static magnetic fields near the surface of mobile phones

**DOI:** 10.1038/s41598-021-98083-9

**Published:** 2021-09-24

**Authors:** L. Zastko, L. Makinistian, A. Tvarožná, F. L. Ferreyra, I. Belyaev

**Affiliations:** 1 Department of Radiobiology, Cancer Research Institute, Biomedical Research Center, University Science Park for Biomedicine, Slovak Academy of Sciences, Bratislava, Slovakia; 2 Department of Physics, Universidad Nacional de San Luis (UNSL), Ejército de los Andes 950, CP5700 San Luis, San Luis Argentina; 3 Instituto de Física Aplicada (INFAP), Universidad Nacional de San Luis (UNSL-CONICET), San Luis, Argentina

**Keywords:** Biophysics, Oncology, Risk factors, Physics

## Abstract

Whether the use of mobile phones (MP) represents a health hazard is still under debate. As part of the attempts to resolve this uncertainty, there has been an extensive characterization of the electromagnetic fields MP emit and receive. While the radiofrequencies (RF) have been studied exhaustively, the static magnetic fields (SMF) have received much less attention, regardless of the fact there is a wealth of evidence demonstrating their biological effects. We performed 2D maps of the SMF at several distances from the screen of 5 MP (models between 2013 and 2018) using a tri-axis magnetometer. We built a mathematical model to fit our measurements, extrapolated them down to the phones’ screen, and calculated the SMF on the skin of a 3D head model, showing that exposure is in the µT to mT range. Our literature survey prompts the need of further research not only on the biological effects of SMF and their gradients, but also on their combination with extremely low frequency (ELF) and RF fields. The study of combined fields (SMF, ELF, and RF) as similar as possible to the ones that occur in reality should provide a more sensible assessment of potential risks.

## Introduction

Given the massive use of mobile phones (MP) and the fact it is still widely debated whether they constitute a health hazard for the users, the association of radiofrequency (RF) electromagnetic fields (EMF) with a diversity of pathologies has been extensively studied. An in-depth review of the subject can be found in the 2016 recommendations issued by the European Academy for Environmental Medicine (EUROPAEM)-EMF working group^1^ (and references therein), where the association of RF EMF with cancer, detrimental effects on the nervous system and on fertility and reproduction; and the, so called, electromagnetic hypersensitivity syndrome were discussed thoroughly. While not necessarily detrimental, it is interesting to note that pulsed RF EMF of 1788 MHz (at a maximum specific absorption rate (SAR) of 0.405 W/kg) were reported to affect the heart rate variability in humans (a proxy to the autonomous nervous system)^2^. Also, many epidemiological studies have been pooled into meta-analyses. For instance, Yang et al.^3^ found positive associations between glioma risk and ipsilateral MP use, while not for contralateral use. While independent meta-analyses including several types of intra-cranial tumors are in accordance with those results^4,5^, Röösli et al.^6^ found no association between glioma risk and MP use. Regarding salivary gland tumors, both conclusions have been reached: no association^4,7^, and association (mild but significant)^8^. Miller et al.^9^ proposed that the International Agency for Cancer Research (IARC) should change the classification of RF EMF from MP and other wireless devices from its current IARC Group 2B (possibly carcinogenic) to Group 1 (carcinogenic), whereas organizations such as the Food and Drugs Administration from the USA^10^ or the Swedish Radiation Safety Authority^11^ have come to the exact opposite conclusion.

Due to all this concern and controversy, it is crucial to exhaustively know the fields MP users are exposed to. Naturally, most of the attention has been laid on the RF part of the spectrum, but also some on the extremely low frequency (ELF) range^12–15^. In contrast, static magnetic fields (SMF) from MP have been studied remarkably less^14,16^, in spite of the fact there is a wealth of evidence demonstrating SMF capability of eliciting biological effects (e.g., on genotoxicity, proliferation of both normal and cancerous cells, modulation of reactive oxygen species, enzymatic activity, stem cell proliferation and fate, repair of DNA lesions, and gene expression)^17–23^. Therefore, in this work, we mapped the SMF from 5 MP near the surface of the screen and up to several cm away from it, with the aim of contributing to a more complete characterization of the fields originated from MP.

## Methods

**Figure 1:**
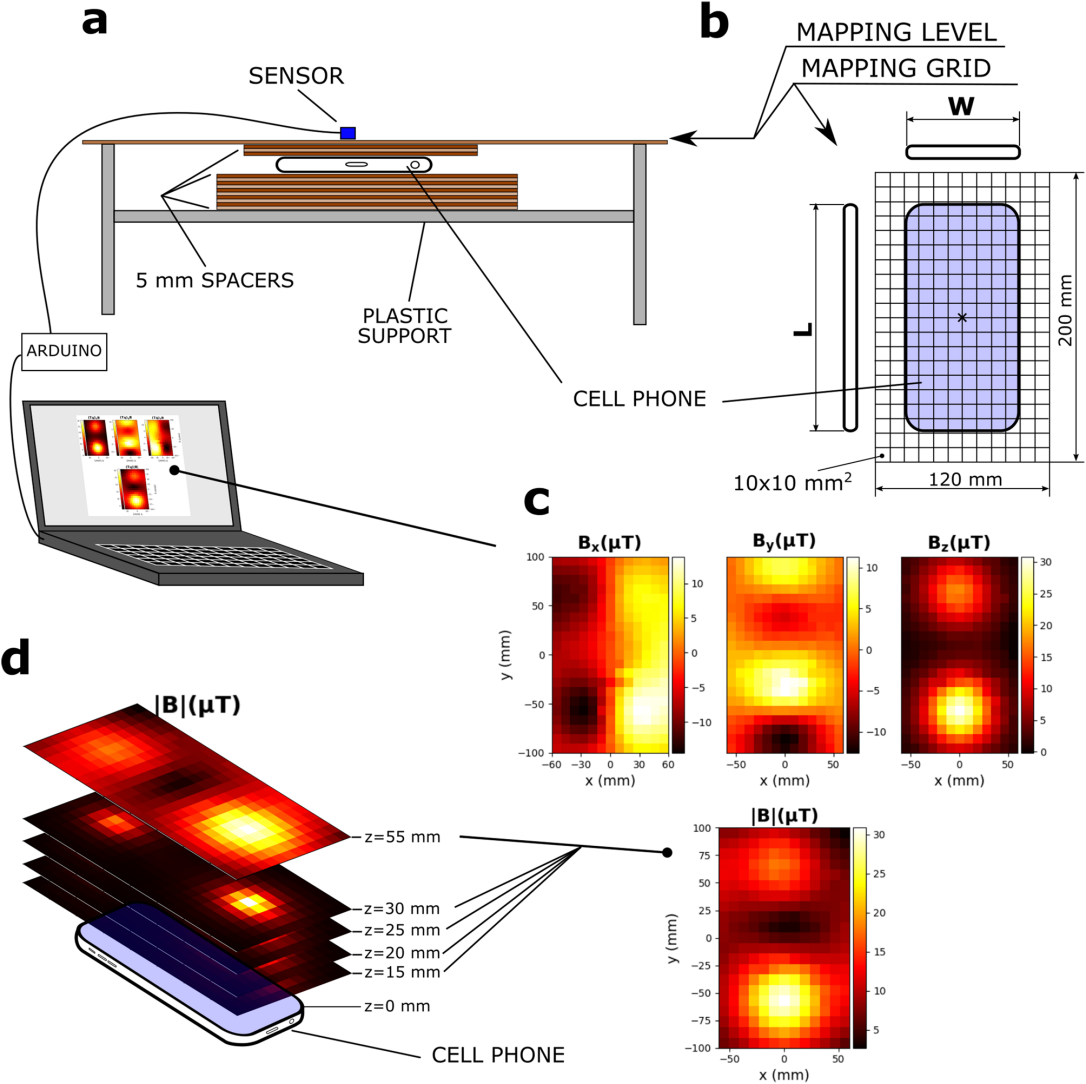
Experimental set up. (**a**) A mobile phone is placed on top of a stack of 5 mm thick PMMA spacers, just below a pane of PMMA which serves as the mapping level. Spacers are also placed above the mobile phone to better guarantee the desired distance and parallelism to the mapping level. On top of the mapping level a grid (**b**) is placed to assist the (manual) placing of a sensor connected to a PC through an ARDUINO board. After all points of the grid are measured with the 3-axis sensor, three maps are generated (one for each of the field’s components, B_x_, B_y_, and B_z_) and the map for the magnitude, |B|, is calculated (**c**). Then one (or more) spacer(s) is(are) removed, so the phone descends 5 mm (or more), increasing its distance from the mapping level, and the procedure of mapping is repeated. Before any phone was placed in site, the ambient background field was mapped. All mappings, for the background and for each value of z (15, 20, 25, 30, and 55 mm) (**d**) were done in triplicate.

Figure 1a shows a sketch of the set up for mapping the SMF: it consisted of a plastic support on top of which the MP to be assessed were placed between stacks of 5 mm thick poly-methyl methacrylate (PMMA) spacers.

The mapping level was defined by a PMMA slab containing a 120 × 200 mm mapping grid with a 10 mm resolution (Fig. 1b). Measurements were taken with a tri-axial magnetometer, HMC5883L (Honeywell, Morris Plains, NJ) connected to a personal computer (PC) through an ARDUINO Leonardo board handled by a custom-written code. The code generated the corresponding B_x_, B_y_, and B_z_ maps delivered by the sensor and calculated the magnitude of the SMF, |B| (Fig. 1c). Then, one or more spacers were removed so the MP descends with respect to the mapping level and mapping was repeated for a different distance, *z*, from the screen of the MP. Five MP models were assessed and for each phone model and value of *z* (15, 20, 25, 30, 55 mm, Fig. 1d), a map was performed in triplicate; the Results section shows the (pixel-by-pixel) average of the SMF magnitude, |B|. Without any MP near the mapping level, the background SMF (geomagnetic) was first mapped (also in triplicate) and then subtracted from all the maps of the MP. Four sources of uncertainty were taken into consideration: (1) repeatability, taken as equal to the standard deviation (SD) of the measurements; (2) the error of zeroing the magnetic sensor (U_zero_), not greater than 2 µT, based on its evaluation upon 180° rotations around the sensor’s axes; (3) calibration error (U_cal_), determined to be of 0.5% after calibration of the sensor against a pair of commercial Helmholtz coils (Model TG-13, UCHIDA, Tokyo, Japan, field generating factor of 7.80 × 10^−4^ T/A) injected with current from a programmable DC power supply (Rigol DP1308A, Beaverton, OR) with a ripple lesser than 500 µA_rms_; and (4) the error (U_grad_) due to the gradient of the field along the *z* direction combined with the uncertainty on the z position, estimated to be between 1.5 and 1.7 mm. A combined uncertainty (U_c_) was calculated adding in quadrature the four said sources of uncertainty (full details of the uncertainty budget are provided in the Supplementary data 1). We did measurements (not full mapping) at the hot spots (HS), i.e., the locations of local maxima in the maps, which were located in the upper and lower parts of the phone’s screen (presumably due to the speaker and the microphone, respectively) in the 6 possible MP modes: (1) simply ON, (2) simply OFF, (3) calling some other phone that is not answering, (4) ringing (MP being called), (5) during an ongoing call, and (6) flight mode (all measurements are in the Supplementary data 1). For 4 of the 5 phones, the highest difference between different modes was not greater than 5%, while not greater than 9% for the remaining model. Based on this assessment, for convenience, we proceeded to map the SMF only with the MP ON (no calls underway, no flight mode). The whole set up was not moved, nor metallic objects were allowed nearby during the measurements. The background AC MF noise in the area of mapping was not greater than 100 nT_rms_.

## Results

**Figure 2:**
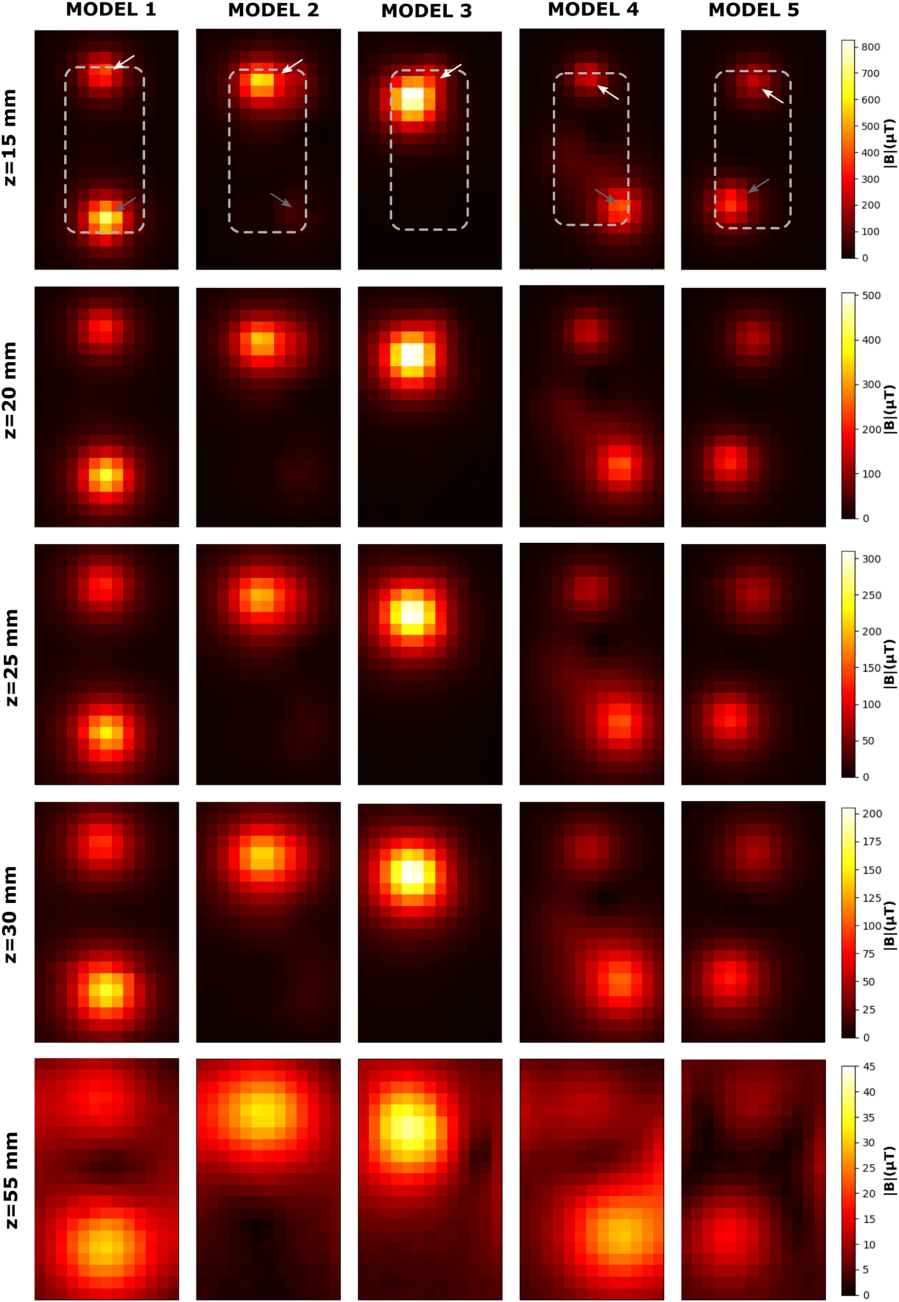
Maps of the magnetic field distribution at different heights above the phone surface (z = 15, 20, 25, 30, 55 mm), for the 5 MP models. Each map shown is the average of three measurements done in a 120 × 200 mm^2^ grid with a pixel size of 10 × 10 mm^2^. Background was subtracted from all maps, so that the actual field originated from the phones is shown.

**Figure 3:**
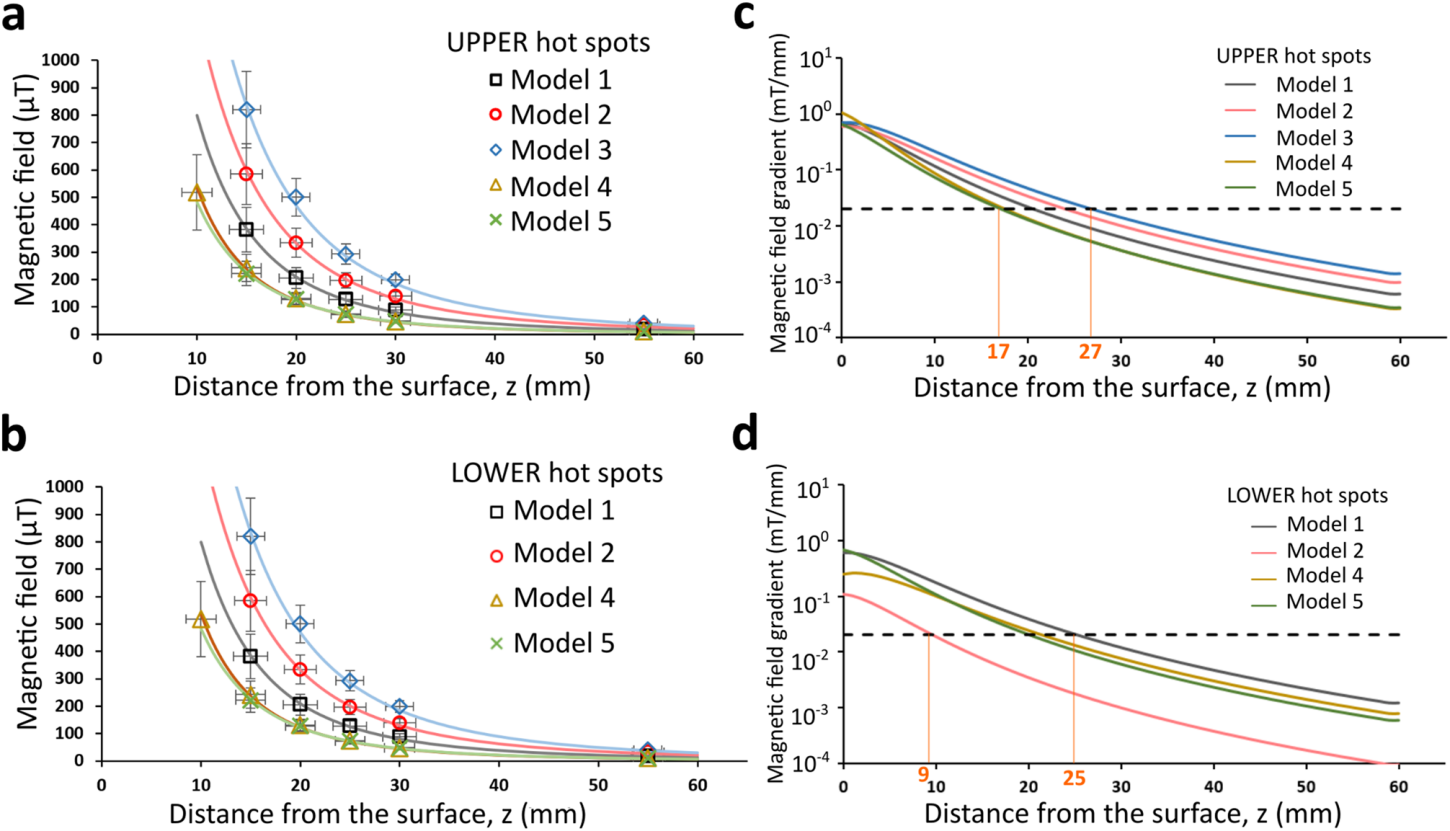
The decay of the field with distance, at the (**a**) upper and (**b**) lower hot spots. Error bars are ± U_c_, the combined uncertainty (see “Methods” for details). Regression curves are according to our SLC-model, which allowed us to calculate the SMF gradients, (**c**,**d**). The dashed line in (**c**) and (**d**) is at 0.02 mT/mm, a threshold of interest reported in the literature (see “Discussion and conclusions”).

Figure 2 presents the 25 maps assessed in this work: from a distance of 15 mm above the MP screen to a distance of 55 mm, for each of the 5 MP. On the first floor of the figure the silhouette of the corresponding MP is represented by dashed lines and an upper and a lower HS is pointed by an arrow (except for MODEL 3, which did not present a lower HS). It can be seen how the fields diffuse and decrease in magnitude as the mapping distance increases: from 820.2 µT at z = 15 mm (MODEL 3) to 3.5 µT at z = 55 mm (MODEL 2). Table 1 gathers the MP dimensions, and details of all the HS; the order of magnitude of the values at *z* = 55 mm is in agreement with the 65 µT reported elsewhere^16^. Figure 3a,b shows the decay of the SMF versus the distance, *z*, from the MP screen (*z* = 15, 20, 25, 30, and 55 mm; z = 10 mm could also be measured in MODEL 4, see Supplementary data 1), while Fig. 3c,d shows the gradients estimated by our SCL-model (see below).

**Table 1:** Dimensions of the cell phones, location on their screen of the magnetic hot spots (HS), and the maximum field measured at each of them.

Model	L (mm)	W (mm)	T (mm)	HS	Location of hot spot (HS)		|B|_max_ @ z = 15 mm (measured) (µT)	|B|_max_ @ z = 55 mm (measured) (µT)
					x (mm)	y (mm)		
1	146.9	70.9	8.4	UPPER	0	70	381.9 ± 81.4	17.5 ± 2.6
				LOWER	0	− 60	705.3 ± 123.9	30.9 ± 3.4
2	143.6	70.9	7.7	UPPER	− 10	60	584.7 ± 111.5	32.4 ± 3.1
				LOWER	30	− 60	59.8 ± 12.8	3.5 ± 2.2
3	142.3	71.0	7.8	UPPER	− 20	50	820.2 ± 139.2	40.2 ± 3.4
				LOWER	–	–	–	–
4	133.9	68.7	7.5	UPPER	0	60	242.3 ± 50.0	11.1 ± 2.3
				LOWER	30	− 50	404.8 ± 72.8	29.6 ± 2.7
5	139.6	69.7	9.1	UPPER	0	60	222.0 ± 43.9	9.2 ± 2.5
				LOWER	− 20	− 50	361.0 ± 63.9	16.4 ± 2.6

Magnetic field values are expressed ± U_c_, the combined uncertainty (see “Methods” for details).

**Figure 4:**
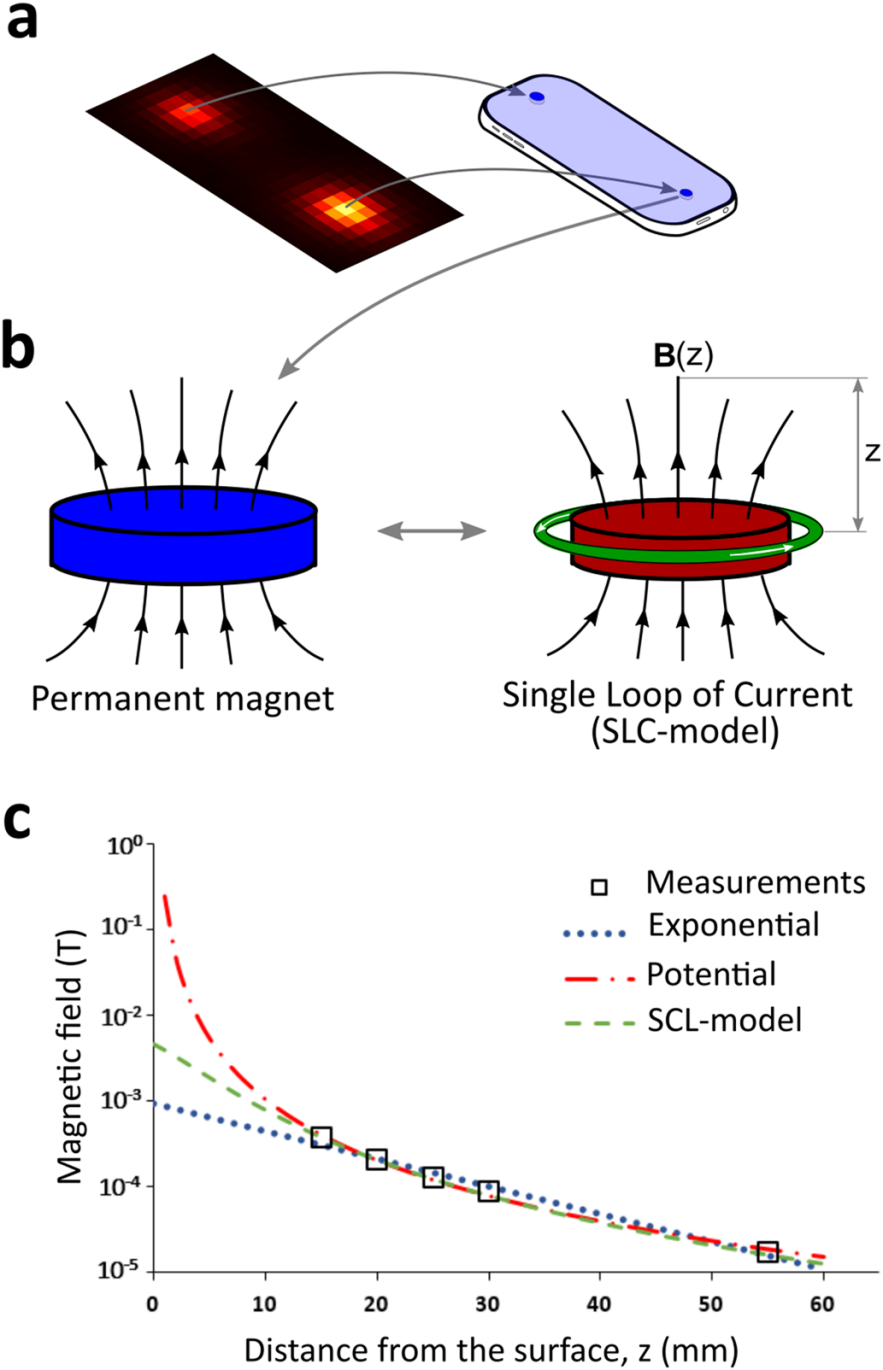
(**a**) Magnetic hot spots are due to built-in permanent magnets of the speaker and microphone of the mobile phones. (**b**) We used an idealization to model a permanent magnet: a simple loop of current (SLC) around a high permeability material. The magnetic field intensity along the magnet axis, **B**(z), is described by Eq. (1). (**c**) Exponential, potential, and SCL-model regressions of our data for the upper hot spot of MODEL 1 illustrate differences between the three regressions.

In order to extrapolate our measurements at the HS down to the screen of the MP, we modeled these sources of SMF (presumably, the MP speaker (upper HS) and microphone (lower HS)) as a button-like cylindrical permanent magnet (Fig. 4a), which in turn can be represented as a single loop of current (SLC) with a magnetic core (Fig. 4b). The MF along the axis of a circular loop is well known^24^ to be a function of distance:

1 $$B\left(z\right)=\frac{{\mu }_{r}{\mu }_{0}I{r}^{2}}{{2\left[{\left(z+T/2\right)}^{2}+{r}^{2}\right]}^{\frac{3}{2}}}$$

Here ${\mu }_{r}$ is the relative permeability of the material of the core (${\mu }_{r}$ = 5000 in our model), ${\mu }_{0}$ is the magnetic permeability of vacuum, $I$ is the current, $r$ is the radius of the current loop, $z$ is the distance from the MP screen and $T$ is the MP thickness (see Table 1). The term T/2 signifies that we assume the SLC is at half the thickness of the MP below the screen. Figure 4c shows our measured data from the upper HS of MODEL 1 fitted to the SCL-model, as well as to an exponential and a potential regression. It is clearly seen that the exponential seems to underestimate the extrapolation at the MP screen (*z* = 0), while the potential simply diverges. Table 2 shows that our SCL-model fits the data clearly better than the exponential (compare values of R^2^) and also better than the potential regression in almost all cases. The extrapolated values of the SMF at the MP screen are roughly around 5 mT (except for the lower HS of MODEL 2, which is below 1 mT, last column of Table 2). This order of magnitude is in good agreement with previously reported values^14^ which, nevertheless, were greater (20 mT, the authors did not report the extrapolation regression model they used). Over the 5 MP models, we estimated SMF intensities of 0.74–6.6 mT and gradients of ~ 0.1 to 1.1 mT/mm on the screen of the MP (Fig. 3c,d).

**Table 2:** Parameters of the regression used to fit the measurements: exponential, potential, and our SCL-model.

Model	Hot spot	Exponential $a{e}^{-bz}$			Potential $a{z}^{-b}$			SLC-model $\frac{{\mu }_{r}{\mu }_{0}I{r}^{2}}{{2\left[{\left(z+T/2\right)}^{2}+{r}^{2}\right]}^\frac{3}{2}}$			
		a = B@0 (mT)	b (mm^-1^)	R^2^	a (µT)	b	R^2^	*R* (mm)	*I* (mA)	R^2^	B@0 (mT)
1	UPPER	0.93	0.074	0.982	250,029	2.369	0.995	8.0	17.2	0.998	4.69
	LOWER	1.77	0.075	0.981	537,816	2.419	0.995	10.0	22.6	0.998	5.56
2	UPPER	1.32	0.069	0.974	262,720	2.236	0.998	9.5	19.8	0.998	5.21
	LOWER	0.13	0.068	0.962	23,115	2.196	0.999	8.0	2.59	0.998	0.74
3	UPPER	2.10	0.073	0.985	537,666	2.350	0.993	10.0	26.0	0.996	6.60
	LOWER	–	–	–	–	–	–	–	–	–	–
4	UPPER	0.77	0.082	0.948	109,264	2.228	0.996	6.0	16.0	0.997	5.11
	LOWER	1.12	0.070	0.948	77,710	1.949	0.997	11.0	11.6	0.998	2.81
5	UPPER	0.58	0.077	0.983	203,089	2.478	0.995	7.0	13.2	0.997	3.49
	LOWER	0.92	0.075	0.985	266,219	2.399	0.994	8.0	17.0	0.997	4.38

The potential fitting predicts B@0 → ∞, so it is not consigned in the table.

**Figure 5:**
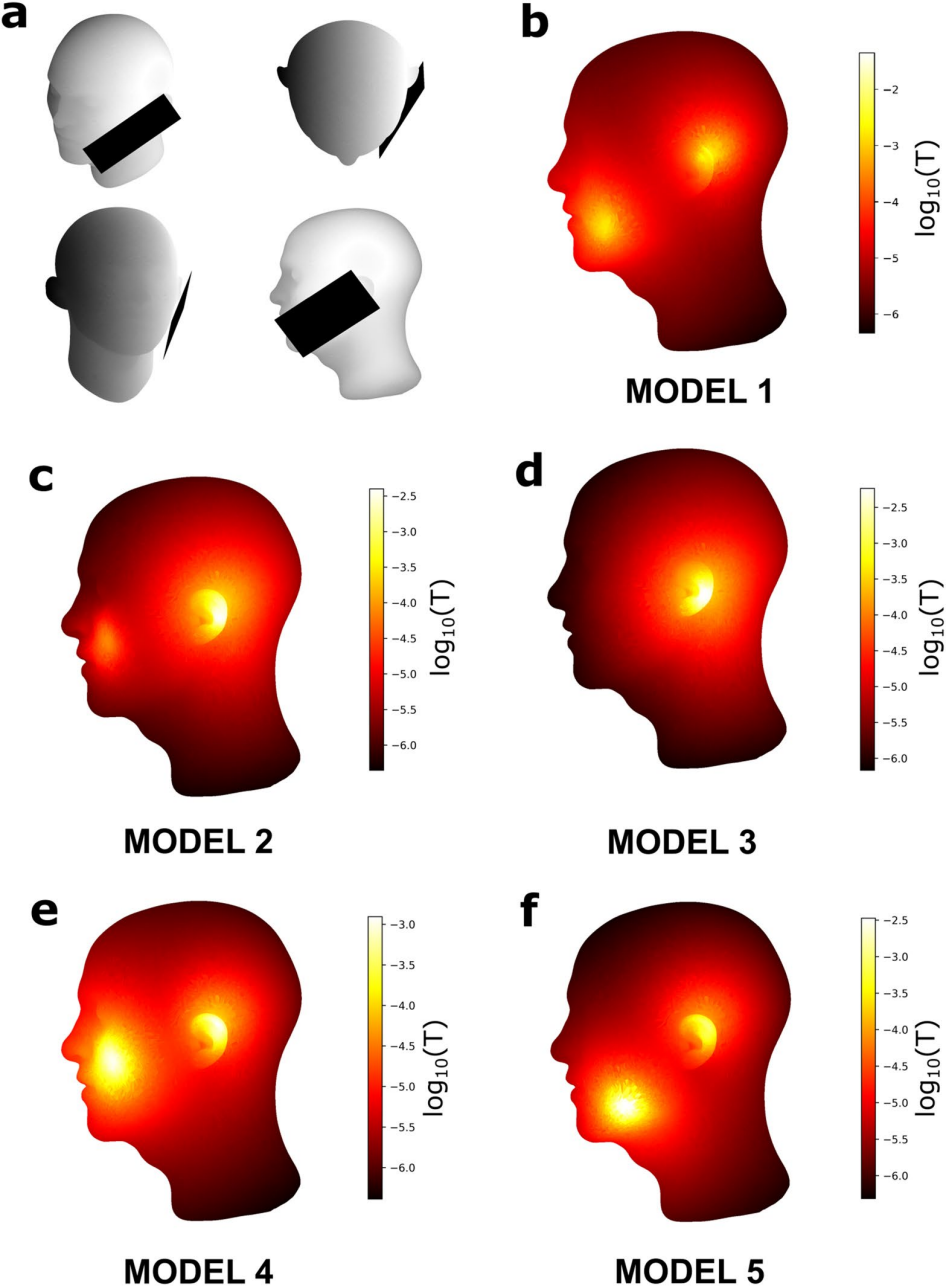
(**a**) Location of the phone (shades of gray are only lighting, not magnetic field data). (**b**–**f**) Magnetic field distribution on the head model according to the simulations (fitted to the measurements).

The agreement of our measurements with the SCL-model encouraged us to use their optimized parameters (the radius and the current, *r* and *I*, Table 2) to calculate the SMF distribution on a model head (obtained from https://www.turbosquid.com/es/3d-model/free/male-head, accessed on 11 Dec 2020) with a MP placed in position typical of an ongoing call (Fig. 5a). Since the SCL-model corresponds to the solution of the Biot-Savart law of magnetostatics, we used the same parameters of the SCL-model as input for a 3D solution of that law on the surface of the head model; using a custom-written Python code already validated elsewhere^25^. The 3D SMF calculations (Fig. 5b–f) allow for a visualization of the field distribution on the skin, where the magnetic HS are clearly devised. It is also shown that due to the rapid decay of the field with distance, more than three orders of magnitudes (µT to mT) affect the MP user at the same time.

## Discussion and conclusions

The maximum SMF estimated in this work (6.6 mT) and others’ (20 mT)^14^ are far below the 400 mT exposure limit for the general public recommended by the International Commission on Non-Ionizing Radiation Protection (ICNIRP)^26^. This might lead to the temptation of completely disregarding SMF as a variable of interest when assessing MP fields. In fact, a recent fact sheet from the Zwiss Federal Office of Public Health^13^ completely omitted any reference to SMF. Nevertheless, the sole capacity of these fields of inducing potentially harmful biological effects such as promotion of the production of reactive oxygen species (ROS)^21^ should be enough to consider them seriously. As for the possible underlying mechanisms of interaction, the one based on magnetite nanoparticles^27^ is of particular interest because these nanoparticles have been found in the human brain^28,29^. Besides the brain, the ear is of an obvious interest for its proximity to the upper HS of MP. Interestingly, magnetic material was reported on the lagenal otoliths of fish and birds^30^ (although this structure is not conserved in mammals). To the best of our knowledge, there have not been studies reporting the search of magnetic materials as constituents of the human inner ear. In this regard, it is to be mentioned that some epidemiological studies found an association between MP use and tinnitus (the perception of a sound in the absence of an external source), while a meta-analysis found no association^31^.

A distinctive feature of the MP SMF reported here is their marked inhomogeneity, which naturally prompts the question of whether the SMF *gradient* is a variable of interest. Notably, this has rarely been studied. By far, most studies evaluate effects of homogeneous fields, and most of the ones that use inhomogeneous fields do not characterize them in detail. A remarkable exception is the work led by Mc Lean in the 1990’s^32,33^. The authors studied the blockade of the firing of action potentials (AP) in neurons extracted from mice upon exposure to extremely well characterized inhomogeneous SMF. They observed an effect of up to 80% at 10 mT and 3.2 mT/mm; 73.4% at 4.7 mT and 0.2 mT/mT; and 41% at 1.4 mT and 0.08 mT/mm; and concluded that gradients above the threshold of ~ 0.02 mT/mm were necessary to observe an effect (they also estimated a minimum necessary field intensity of ~ 0.025 mT). In Fig. 3c,d) the dashed line indicates the 0.02 mT/mm SMF gradient threshold. Our calculated gradients are above that value at distances up to—depending on the MP model—between 17 and 27 mm (9 and 25 mm) from the MP screen at the upper (lower) HS. Since, the average thickness of the skull at the temporal lobe is ~ 3 and 4 mm^34^, our calculations predict that for all 5 MP assessed, the values of the SMF *and its gradient* inside the skull are above the thresholds found by Cavopol et al.^32^ These results (and others^35,36^) strongly suggest the need of further studies controlling both, SMF and SMF gradient, for which special devices have been developed^37^.

Considering that the ICNIRP issued guidelines regarding induced electric fields (EF) due to motion in a SMF^38^, it is worth asking whether they might be relevant in the context of the present work. While using a MP does not involve motion in a SMF (such being the case of a health worker in a magnetic resonance imaging (MRI) facility), the act of taking the MP onto the head implies a “rapid” change of the MF which, in turn, induces an EF. This EF is a transient that exists only while the phone is in motion: as soon as the MP remains in a fixed position onto the head, the time derivative of the magnetic field is zero and so the induced EF. Assuming that the duration of this pulse of EF is in the range of 0.1–1 s (depending on how fast the MP is taken to the ear), and considering that the maximum values of SMF found in this work are in the range of a few mT, we roughly estimate a rate of change of the MF of the order of 1–10 mT/s. This is far below the reference level of 2.7 T/s recommended by the ICNIRP. Therefore, it is extremely unlikely that taking a MP to the head will imply a risk of nerve stimulation, magnetophosphenes, or vertigo (which are, indeed, to be expected upon rapid movement in SMF of several Teslas).

Another implication of our mappings is that given the cylindrical symmetry of the MP SMF and the fact their range inside the head includes the geomagnetic field (GMF) values (25–65 µT) it is likely that, at some point of space, the MP’s MF will have an intensity similar to that of the GMF, but with the opposite direction, hence compensating it. This would mean the realization of zones of hypomagnetic fields (i.e., near-zero fields) inside the head close to the location of the phone’s HS. This might be of relevance given that, upon nulling or shielding of the environmental MF, a diversity of bioeffects have been reported on, e.g., human fibroblasts proliferation, circadian rhythms of the house sparrow, several plants growth, blood analysis parameters of Wistar rats, and eye neural activity of the fruitfly, among many others analyzed in the extensive reviews by Binhi and Prato^39^ and Zhang et al.^40^.

Beyond the fact that effects of SMF on head tissues are worth investigating per se, there seems to be an even less studied, intricate relation between RF EMF effects and SMF. For instance, it has been reported that resonance effects of RF on *E. coli* cells depend on the magnitude of the SMF at the location of RF exposure^41^. This dependence was explained by a model of electron-conformational interactions that also predicts the possible shift of RF resonance frequencies caused by the SMF^42^. Gapeev et al. analyzed effects of RF exposure (41.85–42.1 GHz, frequency increment 50 MHz, power density (PD) 50 μW/cm^2^, for 20 min) performed at various SMF on the synergistic reaction of calcium ionophore A23187 and phorbol ester PMA in the activation of the respiratory burst of peritoneal neutrophils of mice^43^. At a SMF of 50 μT, the authors observed frequency-dependent inhibition of the synergetic reaction with maximal effect at the frequency of 41.95 GHz. In the same frequency range, frequency-dependent activation of the synergetic reaction with a maximal effect at the frequency of 42.0 GHz was found at a SMF of 95 μT. The authors concluded that increasing the SMF from 50 to 95 μT resulted in the inversion of RF effects and the shift of the resonance frequency by 50 MHz. Moreover, these effects of RF at the 41.95 GHz and 42.0 GHz were not found at the SMF of ± 1, 28.3, 75.5 or 117.3 μT, suggesting that the RF effects appear only at specific SMF^43,44^. More recently, Ushakov et al.^45^ exposed *E. coli* cells to RF at the PD of 10^–10^ W/cm^2^ and the frequencies of 51.675, 51.755 and 51.835 GHz. In their study, cells were exposed to RF at various values of SMF within the range of the geomagnetic field: 22, 49, 61, or 90 μT. The authors observed that the RF effects on the conformation of nucleoids depended on the SMF during exposure. The relation of the SMF and RF EMF was also confirmed in the context of magnetoreception^46^, where the orientation of birds with respect to an external SMF was proven to be impaired by the simultaneous exposure to broadband noise RF EMF (50 kHz–5 MHz^47^, and ~ 2 kHz to ~ 9 MHz^48^). The orientation of yearling snapping turtles was also reported to be affected by weak, narrowband RF EMF (1.43 MHz, 30–52 nT)^49^. Complementary to experiments with broadband noise and narrow band RF, it would be desirable the realization of experiments with exposure systems testing real telecommunication signals^50–52^ combined with (non-homogeneous) SMF.

Our measurements and calculations, along with the preceding discussion of the literature, prompt the need of further studies of the biological effects of the SMF and RF EMF generated by MP. We suggest special emphasis should be put into (1) inhomogeneous SMF (with a detailed characterization of both, intensities and gradients), and (2) combination of SMF (homogeneous and inhomogeneous) with RF EMF, with a fine sweeping of the parameters within the range of typical exposures (in opposition to testing single combinations). In even more complicated experiments, ELF EMF should also be considered^12^ since their relationship with SMF have been long known^53^. Lastly it is worth mentioning that substantial reduction of the SMF from MP could be accomplished by replacing the standard electrodynamic loudspeakers and microphones (which include a permanent magnet) by their capacitive counterparts (which do not).

## Supplementary Information

Supplementary Information 1.

## Data Availability

The datasets generated during and/or analyzed during the current study are available from the corresponding author on reasonable request.
