# Supplementary material for: Mapping of static magnetic fields near the surface of mobile phones
Source: Sci Rep. 2021 Sep 24;11:19002. doi: 10.1038/s41598-021-98083-9 (PMC8463716; doi:10.1038/s41598-021-98083-9)
Supplement: Supplementary file 1 — Supplementary Information 1. [file 41598_2021_98083_MOESM1_ESM.pdf]

SUPPLEMENTARY MATERIAL FOR

Mapping of static magnetic fields near the surface of mobile phones

L. Zastko[1], L. Makinistian[2,3], A. Tvarožná[1], F. L. Ferreyra[2], I. Belyaev[1]

[1] Department of Radiobiology, Cancer Research Institute, Biomedical Research Center, University Science Park for Biomedicine, Slovak Academy of Sciences, Bratislava, Slovakia
[2] Department of Physics, Universidad Nacional de San Luis (UNSL), San Luis, Argentina
[3] Instituto de Física Aplicada (INFAP), Universidad Nacional de San Luis (UNSL)-CONICET, San Luis, Argentina

MEASUREMENTS FOR DIFFERENT MODES

LOWER HOT SPOT (microphone)						
	Release	2016	2018	2016	2014	2013
	Brand & Model	HTC Desire 650	iPhone XS	SAMSUNG Galaxy J3	HUAWEI Honor 6	LG Nexus 4
**Mode**	**n**	**MODEL 1**	**MODEL 2**	**MODEL 3**	**MODEL 4**	**MODEL 5**
**ON**	**1**	658.3	109.3	HSNI*	355.4	414.0
	**2**	646.8	107.6	HSNI*	357.1	409.2
	**3**	648.4	108.1	HSNI*	352.0	412.7
	**AVG**	**651.2**	**108.3**	HSNI*	**354.8**	**412.0**
**OFF**	**1**	666.5	112.5	HSNI*	364.2	421.9
	**2**	667.1	113.1	HSNI*	363.8	419.5
	**3**	665.9	112.4	HSNI*	364.4	420.8
	**AVG**	**666.5**	**112.7**	HSNI*	**364.1**	**420.7**
**Calling**	**1**	645.2	115.4	HSNI*	351.1	410.3
	**2**	646.8	114.9	HSNI*	349.5	410.9
	**3**	648.4	115.2	HSNI*	350.7	411.0
	**AVG**	**646.8**	**115.2**	HSNI*	**350.4**	**410.7**
**Being called**	**1**	672.4	108.9	HSNI*	367.4	415.8
	**2**	673.0	109.1	HSNI*	367.6	416.2
	**3**	674.8	109.3	HSNI*	367.9	416.7
	**AVG**	**673.4**	**109.1**	HSNI*	**367.6**	**416.2**
**On-going call**	**1**	665.5	105.5	HSNI*	358.2	420.5
	**2**	659.9	105.9	HSNI*	358.4	421.4
	**3**	668.9	105.9	HSNI*	358.7	420.9
	**AVG**	**664.8**	**105.2**	HSNI*	**358.4**	**420.9**
**Flight mode**	**1**	650.2	107.8	HSNI*	354.4	407.9
	**2**	654.3	108.2	HSNI*	354.1	409.5
	**3**	652.7	108.1	HSNI*	355.6	407.8
	**AVG**	**652.4**	**108.0**	HSNI*	**354.7**	**408.4**
**Max difference of AVGs (%)**		**4.0**	**8.7**	**-**	**4.7**	**3.0**

***Hot Spot Not Identified, MAX, MIN**

UPPER HOT SPOT (speaker)						
	Release	2016	2018	2016	2014	2013
	Brand & Model	HTC Desire 650	iPhone XS	SAMSUNG Galaxy J3	HUAWEI Honor 6	LG Nexus 4
**Mode**	**n**	**MODEL 1**	**MODEL 2**	**MODEL 3**	**MODEL 4**	**MODEL 5**
ON	1	324.1	529.1	877.4	200.5	274.7
	2	328.2	529.9	874.5	200.7	275.0
	3	330.4	529.6	873.8	195.3	274.2
	AVG	**327.6**	**529.5**	**875.2**	**198.8**	**274.6**
OFF	1	326.2	534.2	880.3	199.3	277.7
	2	326.1	534.4	880.9	198.8	278.0
	3	327.0	535.1	879.5	198.7	275.3
	AVG	**326.4**	**534.6**	**880.2**	**198.9**	**277.0**
Calling	1	325.3	525.7	875.3	201.5	275.3
	2	325.4	526.0	874.9	202.3	275.4
	3	326.1	525.1	874.8	199.5	275.9
	AVG	**325.6**	**525.6**	**875.0**	**201.1**	**275.5**
Being called	1	334.1	538.2	883.1	196.3	280.6
	2	334.8	538.9	883.5	197.2	280.7
	3	335.0	538.0	882.9	196.5	281.1
	AVG	**334.6**	**538.4**	**883.2**	**196.7**	**280.8**
On-going call	1	336.2	528.4	876.1	196.3	269.3
	2	336.1	527.9	876.9	196.3	267.9
	3	337.0	528.3	876.9	196.8	269.9
	AVG	**336.4**	**528.2**	**876.6**	**196.5**	**269.0**
Flight mode	1	319.9	533.3	879.2	205.1	271.8
	2	320.1	532.9	878.9	204.8	272.0
	3	319.8	533.7	879.5	204.7	272.1
	AVG	**319.9**	**533.3**	**879.2**	**204.9**	**272.0**
**Max difference of AVGs (%)**		**4.9**	**2.4**	**0.7**	**4.1**	**4.2**

MAX, MIN

UNCERTAINTY BUDGET

Four sources of uncertainty were taken into consideration: 1) repeatability, taken as equal to the standard deviation (SD) of the measurements (done in triplicate); 2) the error of zeroing the magnetic sensor (U_{zero}), not greater than 2 µT, based on its evaluation upon 180º rotations around the sensor's axes; 3) calibration error (U_{cal}), determined to be of 0.5% after calibration of the sensor against a pair of commercial Helmholtz coils (Model TG-13, UCHIDA, Tokyo, Japan, field generating factor of 7.80 x 10^{-4} T/A) injected with current from a programmable DC power supply (Rigol DP1308A, Beaverton, OR) with a ripple lesser than 500 μA_{rms}; and 4) the error due to the gradient of the field along the *z* direction (U_{grad}) combined with the uncertainty on the z position (U_z) of the magnetic HS, estimated to be between 1.5 and 1.7

mm. A combined uncertainty (U_c) was calculated adding in quadrature the four said sources of uncertainty: $U_c^2 = SD^2 + U_{zero}^2 + U_{cal}^2 + U_{grad}^2$.

The parallelism of the phone and the mapping plane was better than 1°, hence the uncertainty regarding z (horizontal error bars in Figures 3 a) and b) of the manuscript) was U_z = dist*sin(1°), where "dist" is the distance from the hot spot to the center of the phone. The remarkably low uncertainty on the angle (better than 1°) was accomplished placing acrylic spacers air-tight one onto the others between the mobile phone and the mapping level (Figure 1a) of the manuscript). As for the position of the sensor, extreme attention was put to making it coincide to the grid within 0.5 mm (we estimate an extra uncertainty of 0.5 mm due to the unknown exact position of the sensor itself within the plastic encapsulation that contains it).

Here is a summary of the uncertainty budget and the calibration of the sensor:

Offest correction error (Uzero)	2	µT	Magnetic field sensor calibration against UCHIDA Helmholtz coils (780 uT/A)				
Calibration error (Ucal)	0,5 % / 100 x B (µT)	µT		uT/A before calibration	uT/A after calibration	Ideal uT/A	%error
Repeatability (Ur)	SD	µT	Bx	766,0	776,4	780	0,5
Uncertainty due to gradient (Ugrad)	grad (µT/mm) x Uz (mm)	µT	By	772,3	779,7	780	0,0
SD: Standard Deviation			Bz	771,7	779,6	780	0,1
						%error added in quadrature	0,5

Next, all values shown in Table 1 and Figures 3a) and b), including the combined uncertainty with its components discriminated. For the UPPER HS:

	HS x position (mm)	HS y position (mm)	dist_to_center (mm)	z position uncertainty, Uz (mm)
MODEL 1	0	70	70,0	1,7
MODEL 2	-10	60	60,8	1,6
MODEL 3	-20	50	53,9	1,4
MODEL 4	0	60	60,0	1,5
MODEL 5	0	60	60,0	1,5

	HORIZONTAL ERROR BARS (UPPER HS)				
z (mm)	MODEL 1	MODEL 2	MODEL 3	MODEL 4	MODEL 5
10	-	-	-	1,5	-
15	1,7	1,6	1,4	1,5	1,5
20	1,7	1,6	1,4	1,5	1,5
25	1,7	1,6	1,4	1,5	1,5
30	1,7	1,6	1,4	1,5	1,5
55	1,7	1,6	1,4	1,5	1,5

	MODEL 1 (UPPER)							
**z (mm)**	**Field AVG (spk) (μT)**	**Field SD (μT)**	**Estimated gradient (μT/mm)**	**Uzero (μT)**	**Ucal (μT)**	**Ugrad (μT)**	**Combined uncertainy, Uc (μT)**	**Combined uncertainy (%)**
**15**	381,9	6,2	47,1	2,0	1,9	81,1	81,4	21,3
**20**	205,4	2,8	21,7	2,0	1,0	37,4	37,5	18,3
**25**	127,5	2,5	11,2	2,0	0,6	19,3	19,6	15,3
**30**	88,5	1,1	6,3	2,0	0,4	10,8	11,1	12,5
**55**	17,5	0,9	0,8	2,0	0,1	1,4	2,6	14,8

	MODEL 2 (UPPER)							
**z (mm)**	**Field AVG (spk) (μT)**	**Field SD (μT)**	**Estimated gradient (μT/mm)**	**Uzero (μT)**	**Ucal (μT)**	**Ugrad (μT)**	**Combined uncertainy, Uc (μT)**	**Combined uncertainy (%)**
**15**	584,7	20,9	70,1	2,0	2,9	109,5	111,5	19,1
**20**	333,8	3,6	33,6	2,0	1,7	52,5	52,7	15,8
**25**	196,3	2,3	17,7	2,0	1,0	27,6	27,8	14,2
**30**	139,1	2,4	10,1	2,0	0,7	15,8	16,1	11,6
**55**	32,4	1,1	1,3	2,0	0,2	2,0	3,1	9,5

	MODEL 3 (UPPER)							
**z (mm)**	**Field AVG (spk) (μT)**	**Field SD (μT)**	**Estimated gradient (μT/mm)**	**Uzero (μT)**	**Ucal (μT)**	**Ugrad (μT)**	**Combined uncertainy, Uc (μT)**	**Combined uncertainy (%)**
**15**	820,2	15,6	96,0	2,0	4,1	138,2	139,2	17,0
**20**	500,6	12,4	46,9	2,0	2,5	67,5	68,7	13,7
**25**	292,9	7,4	25,0	2,0	1,5	36,0	36,8	12,6
**30**	198,7	4,1	14,3	2,0	1,0	20,6	21,1	10,6
**55**	40,2	1,0	1,8	2,0	0,2	2,6	3,4	8,5

	MODEL 4 (UPPER)							
**z (mm)**	**Field AVG (spk) (μT)**	**Field SD (μT)**	**Estimated gradient (μT/mm)**	**Uzero (μT)**	**Ucal (μT)**	**Ugrad (μT)**	**Combined uncertainy, Uc (μT)**	**Combined uncertainy (%)**
**10**	518,0	18,3	87,7	2,0	2,6	135,7	136,9	26,4
**15**	242,3	11,7	31,4	2,0	1,2	48,6	50,0	20,6
**20**	131,3	4,8	13,5	2,0	0,7	20,9	21,5	16,4
**25**	73,6	2,9	6,7	2,0	0,4	10,4	11,0	14,9
**30**	47,9	2,0	3,7	2,0	0,2	5,7	6,4	13,3
**55**	11,1	1,0	0,4	2,0	0,1	0,6	2,3	20,2

	MODEL 5 (UPPER)							
**z (mm)**	**Field AVG (spk) (μT)**	**Field SD (μT)**	**Estimated gradient (μT/mm)**	**Uzero (μT)**	**Ucal (μT)**	**Ugrad (μT)**	**Combined uncertainy, Uc (μT)**	**Combined uncertainy (%)**
**15**	222,0	1,8	28,3	2,0	1,1	43,8	43,9	19,8
**20**	127,8	4,7	12,8	2,0	0,6	19,8	20,5	16,0
**25**	72,8	1,7	6,6	2,0	0,4	10,2	10,5	14,5
**30**	48,4	2,0	3,7	2,0	0,2	5,7	6,4	13,2
**55**	9,2	1,2	0,5	2,0	0,0	0,8	2,5	27,0

	VERTICAL ERROR BARS (UPPER HS)				
**z (mm)**	**MODEL 1**	**MODEL 2**	**MODEL 3**	**MODEL 4**	**MODEL 5**
**10**	-	-	-	136,9	
**15**	81,4	111,5	139,2	50,0	43,9
**20**	37,5	52,7	68,7	21,5	20,5
**25**	19,6	27,8	36,8	11,0	10,5
**30**	11,1	16,1	21,1	6,4	6,4
**55**	2,6	3,1	3,4	2,3	2,5

For the LOWER HS:

	HS x position (mm)	HS y position (mm)	dist_to_center (mm)	z position uncertainty (mm)
MODEL 1	0	60	60,0	1,5
MODEL 2	30	-60	67,1	1,7
MODEL 4	30	-50	58,3	1,5
MODEL 5	-20	-50	53,9	1,4

HORIZONTAL ERRORBARS (LOWER HS)				
z (mm)	MODEL 1	MODEL 2	MODEL 4	MODEL 5
10	-	-	1,5	-
15	1,5	1,7	1,5	1,4
20	1,5	1,7	1,5	1,4
25	1,5	1,7	1,5	1,4
30	1,5	1,7	1,5	1,4
55	1,5	1,7	1,5	1,4

	MODEL 1 (LOWER)							
z (mm)	Field AVG (spk) (µT)	Field SD (µT)	Estimated gradient (µT/mm)	Uzero (µT)	Ucal (µT)	Ugrad (µT)	Combined uncertainy (µT)	Combined uncertainy (%)
15	705,3	9,4	79,8	2,0	3,5	123,5	123,9	17,6
20	397,9	2,6	39,2	2,0	2,0	60,6	60,8	15,3
25	224,6	1,3	20,9	2,0	1,1	32,3	32,4	14,4
30	161,4	1,8	12,0	2,0	0,8	18,6	18,8	11,6
55	30,9	1,2	1,6	2,0	0,2	2,5	3,4	11,0

	MODEL 2 (LOWER)							
z (mm)	Field AVG (spk) (µT)	Field SD (µT)	Estimated gradient (µT/mm)	Uzero (µT)	Ucal (µT)	Ugrad (µT)	Combined uncertainy (µT)	Combined uncertainy (%)
15	59,8	2,0	7,5	2,0	0,3	12,5	12,8	21,5
20	33,0	0,8	3,4	2,0	0,2	5,7	6,1	18,4
25	19,3	0,9	1,8	2,0	0,1	3,0	3,7	19,3
30	13,3	0,9	1,0	2,0	0,1	1,7	2,8	20,8
55	3,5	0,8	0,1	2,0	0,0	0,2	2,2	61,9

	MODEL 4 (LOWER)							
z (mm)	Field AVG (spk) (µT)	Field SD (µT)	Estimated gradient (µT/mm)	Uzero (µT)	Ucal (µT)	Ugrad (µT)	Combined uncertainy (µT)	Combined uncertainy (%)
10	807,0	40,9	99,8	2,0	4,0	151,5	157,0	19,5
15	404,8	8,9	47,6	2,0	2,0	72,2	72,8	18,0
20	245,2	3,4	24,0	2,0	1,2	36,4	36,7	14,9
25	149,3	3,0	13,0	2,0	0,7	19,7	20,1	13,4
30	105,2	1,8	7,5	2,0	0,5	11,4	11,7	11,1
55	29,6	0,9	1,0	2,0	0,1	1,5	2,7	9,0

	MODEL 5 (LOWER)							
z (mm)	Field AVG (spk) (µT)	Field SD (µT)	Estimated gradient (µT/mm)	Uzero (µT)	Ucal (µT)	Ugrad (µT)	Combined uncertainy (µT)	Combined uncertainy (%)
15	361,0	8,0	44,0	2,0	1,8	63,4	63,9	17,7
20	207,1	5,7	20,4	2,0	1,0	29,4	30,0	14,5
25	125,9	2,9	10,6	2,0	0,6	15,3	15,7	12,5
30	83,9	1,4	6,0	2,0	0,4	8,6	9,0	10,7
55	16,4	1,2	0,8	2,0	0,1	1,2	2,6	15,9

	VERTICAL ERROR BARS (LOWER HS)			
z (mm)	MODEL 1	MODEL 2	MODEL 4	MODEL 5
10	-	-	157,0	-
15	123,9	12,8	72,8	63,9
20	60,8	6,1	36,7	30,0
25	32,4	3,7	20,1	15,7
30	18,8	2,8	11,7	9,0
55	3,4	2,2	2,7	2,6
